# Psychological capital, perceptions of clinical stressors, and bullying experiences among Chinese nursing students during clinical practice: a latent profile and mediation analysis

**DOI:** 10.3389/fpubh.2026.1915028

**Published:** 2026-08-27

**Authors:** Fangfei Zhao, Hongchen Zhang, Ke Hui, Haixin Zheng, Zhihui He

**Affiliations:** School of Nursing, Lanzhou University, Lanzhou, China

**Keywords:** bullying experiences, clinical practice, latent profile analysis, mediation analysis, nursing students, perceptions of clinical stressors, psychological capital

## Abstract

**Background:**

Bullying experiences during clinical practice threaten nursing students' learning and professional development. However, existing studies mainly focused on variable-centered associations, with limited exploration of heterogeneous subgroups from a person-centered perspective. This study aimed to examine the mediating role of perceptions of clinical stressors (PCS) in the relationship between psychological capital (PsyCap) and bullying experiences, while identifying latent profiles of bullying experiences. Recognizing distinct subgroups and clarifying influencing mechanisms can help provide tailored educational interventions.

**Methods:**

A multicenter cross-sectional study was conducted from September 2024 to May 2025. A total of 578 nursing students undergoing clinical internships at six general hospitals in Gansu Province, China, were enrolled in the survey. This study included general information questionnaires, the Psychological Capital Questionnaire, the Perceptions of Clinical Stressors Scale, and the Bullying Behaviors in Nursing Education Scale. Mediation analysis and latent profile analysis were carried out.

**Results:**

(1) Three distinct latent bullying experiences profiles were identified: the low bullying group (29.2%), the high bullying group (27.9%), and the moderate bullying group (42.9%). (2) PsyCap was significantly negatively associated with bullying experiences (*r* = −0.635, *p* < 0.001). (3) PCS partially mediated the relationship (*ab*= −0.248, *p* < 0.001) between PsyCap and bullying experiences, with a proportion mediated of 43.12%.

**Conclusions:**

Bullying experiences among nursing students are heterogeneous and embedded in stressful clinical learning environments. PCS and PsyCap are key factors associated with this heterogeneity, with PCS acting as a partial mediator. Nursing educators should implement targeted, profile-specific interventions to mitigate the impact of bullying experiences.

## 1 Introduction

Clinical practice constitutes a core component of nursing education, enabling students to integrate theoretical knowledge with hands-on patient care while developing professional competence and identity. Nevertheless, the hierarchical ward structures and unfamiliar working environments leave nursing students vulnerable to bullying during clinical rotations. A recent meta-analysis reported that approximately 65.6% of nursing students experienced bullying during clinical placements, far exceeding the prevalence among registered nurses (1–3). This high prevalence underscores bullying as a pervasive and urgent problem in nursing education worldwide.

Bullying in clinical practice refers to repeated, unwanted behaviors intended to humiliate, intimidate, or undermine individuals, typically within relationships characterized by power imbalances (4). In nursing education, bullying may manifest as social exclusion, academic aggression, personal attacks, or direct negative behaviors perpetrated by clinical instructors, staff nurses, or peers (5). Exposure to bullying during clinical practice negatively affects nursing students' psychological well-being, self-esteem (6), and professional confidence (7), hindering their learning and transition into competent nurses (8). Qualitative evidence has revealed that students subjected to bullying experience heightened psychological stress (9) and adverse emotional responses (10), which may impact long-term professional values and career intentions (11). Bullying may also impair clinical performance, decision-making, and attention to patient safety, potentially increasing the risk of medical errors and compromising care quality (12). Given global nursing shortages, the potential loss of future nurses due to negative clinical experiences poses a serious challenge to healthcare systems worldwide (13).

Within the Chinese nursing education framework, the duration and intensity of clinical training vary considerably by educational level (14). Junior college and undergraduate nursing students are generally required to complete more than 10 months of continuous clinical placement before graduation, whereas Master of Nursing Specialist (MNS) programs mandate substantially longer clinical training periods, often exceeding 18 months (15). During this period, students are expected to work under close supervision while progressively assuming responsibilities comparable to those of staff nurses (16). Such prolonged clinical placements, combined with students' dual roles as learners and frontline care providers, may create a structural vulnerability that heightens their susceptibility to bullying experiences. This highlights the need to identify individual protective resources that may buffer against such experiences.

Psychological capital (PsyCap) comprises four dimensions: self-efficacy, hope, resilience, and optimism (17), which have been linked to more favorable psychological outcomes and reduced vulnerability to negative experiences among nursing students (18). Conservation of Resources (COR) theory posits that individuals strive to obtain, retain, and protect valuable resources, and that resource loss triggers stress and adverse outcomes (19). In the clinical training context, nursing students face multiple resource drains, including time constraints, role ambiguity, and limited support from clinical instructors. Sufficient PsyCap empowers individuals to cope with occupational stress and adversity effectively by replenishing depleted resources and preventing further loss (20). According to COR theory, PsyCap functions as a valuable personal resource that compensates for resource depletion. The theory further suggests that individuals with high PsyCap can be better equipped to cope with bullying experiences.

In addition, theoretical frameworks of workplace bullying suggest that bullying is a multifactorial process shaped by individual, social, and organizational factors (21). Within this framework, contextual stressors such as inadequate teaching practices and limited learning resources contribute to negative learning experiences and may heighten students' risk of bullying experiences (22). Drawing on COR theory, prolonged stressors continuously deplete students' resources, weakening their capacity to cope with negative situations and thereby increasing their susceptibility to bullying experiences. Perceptions of clinical stressors (PCS) reflect individuals' subjective appraisal of environmental demands and their perceived coping capacity. The mediating role of PCS between personal resources and bullying experiences has been validated among nursing students (23). However, the specific pathways through which PCS influences bullying experiences, and whether distinct subgroups of nursing students exhibit different patterns of bullying experiences, remain underexplored.

Latent profile analysis (LPA) is a person-centered statistical approach that uses probabilistic modeling to identify unobserved subpopulations based on patterns of responses across continuous variables (24). Existing latent-profile studies on workplace bullying mainly focus on registered nurses (25); however, few investigations have targeted nursing students, who report markedly higher prevalence of bullying experiences. This study used LPA to identify bullying experience profiles among nursing students during clinical practice, and further examined differences in sociodemographic variables, PsyCap, and PCS across these profiles. By uncovering this hidden heterogeneity, we aimed to provide evidence for targeted interventions that can improve clinical learning environments, strengthen student support systems, enhance professional retention, and ultimately contribute to a sustainable nursing workforce. Based on the above rationale, the following hypotheses were proposed:

H1: Distinct profiles of bullying experiences can be identified among nursing students using LPA;

H2: PsyCap is significantly associated with bullying experiences;

H3: PCS mediates the relationship between PsyCap and bullying experiences.

## 2 Methods

### 2.1 Design

This cross-sectional study adhered to the STROBE reporting guidelines and employed LPA to identify distinct profiles of bullying experiences among nursing students, as well as their associated factors.

### 2.2 Setting and participants

Nursing students participating in clinical internships at six general hospitals in Gansu Province, China, were recruited from September 2024 to May 2025. According to the Chinese hospital rating system, all six facilities are tertiary first-class, the highest rating category. The inclusion criteria comprised: (1) having completed more than 3 months of clinical internship; (2) voluntary participation with informed consent; (3) being full-time nursing students (junior college, undergraduate, or postgraduate students). Exclusion criteria included: (1) students on leave or withdrawn during the survey period; (2) incomplete questionnaire responses.

### 2.3 Data collection

This study employed convenience sampling via the Chinese online survey platform Questionnaire Star. Trained investigators from the six hospitals were provided with the survey link and standardized instructions before data collection. The investigators were advised to use WeChat, a Chinese instant messaging application, to transmit the link to the participants after determining their eligibility. Participation was voluntary and participants could withdraw at any time without repercussions. The online survey included three sections: the questionnaires, an explanation of the study's objectives, and a choice to agree to participate. All questions were mandatory, and each IP address was allowed to submit only once. Survey data were stored in a password-protected electronic file.

A total of 632 responses were received. After rigorous screening, 54 invalid responses were excluded, resulting in 578 valid questionnaires for the final analysis. Methodological recommendations suggest that a minimum of 50 participants per latent class is required to ensure reliable model selection in LPA (26). Given that 3 to 5 classes were anticipated, the minimum sample size was estimated at approximately 250. The final sample of 578 substantially exceeds this requirement.

### 2.4 Instruments

#### 2.4.1 General information questionnaire

To investigate the characteristics of students who experienced higher levels of bullying during clinical practice, we considered potential sociodemographic factors, including age, gender, education background, internship duration, and character features (assessed by a single self-report item: “How would you describe your personality?” with response options: introverted or extroverted), and relationship with instructors.

#### 2.4.2 Perceptions of clinical stressors

The Nursing Students' Perceptions of Clinical Stressors Scale (NSPCSS) was developed by Rafati et al. (27) and translated into Chinese by Zhang et al. (28). It comprises 30 items across six dimensions: instructor's limited clinical competence, inadequate clinical environment, interns' inadequate knowledge and skills, inefficient clinical teaching planning, instructors' inappropriate teaching behaviors, and concerns about nursing career characteristics. Items are rated on a five-point Likert scale, where 1 represents “never” and 5 represents “always”, with higher scores reflecting high levels of PCS. The original questionnaire shows good reliability, with a Cronbach's α of 0.945. In this study, the Cronbach's α is 0.950, confirming good reliability.

#### 2.4.3 Psychological capital

The Psychological Capital Questionnaire (PCQ) was originally developed by Luthans (29), and was later adapted by Zhou (30) to the Chinese context to measure PsyCap among nursing students. The questionnaire is divided into four dimensions and consists of 20 items: self-efficacy (six items), hope (six items), resilience (five items), and optimism (three items). Each item adopts a six-point Likert scale, with scores ranging from 1 (“strongly disagree”) to 6 (“strongly agree”). The total score ranges from 20 to 120, with higher scores reflecting higher levels of psychological capital. The Cronbach's α of the original scale is 0.923. In this study, the Cronbach's α is 0.952, indicating good reliability.

#### 2.4.4 Bullying experiences

The Bullying Behaviors in Nursing Education Scale (BBNE) was originally developed by Cerit et al. (31). For the current study, we adopted the Chinese linguistic version translated and culturally validated by Wang et al. (32). The scale is a self-reported instrument and includes four dimensions: educational environment isolation behaviors (four items), academic aggressive behaviors (four items), attack on personality (six items), and direct negative behaviors (four items), with a total of 18 items. Items are rated on a five-point Likert scale (0 = never to 4 = always). Higher scores indicate more frequent exposure to bullying experiences (33). The original scale demonstrated good reliability, with a Cronbach's α of 0.921. In this study, the Cronbach's α is 0.963, indicating satisfactory reliability.

### 2.5 Data analysis

IBM SPSS Statistics 27.0 (IBM Corporation, Location: Armonk, NY, USA) was employed for data entry and analysis; no missing values were present. Normality was assessed using the Shapiro–Wilk test, histograms, and Q-Q plots. Continuous variables were presented as mean ± standard deviation (SD), whereas categorical variables were presented as frequencies and percentages. LPA based on the four dimensions of the BBNE was performed using Mplus 8.11 (Muthén & Muthén, Location: Los Angeles, CA, USA). The evaluation criteria were as follows: Entropy, with values above 0.80 indicating high classification accuracy; lower values of the Akaike Information Criterion (AIC), Bayesian Information Criterion (BIC), and adjusted Bayesian Information Criterion (aBIC) indicated better model fit. Additionally, the k-class model was deemed to have a better fit than the (k-1)-class model if the *p*-values for the Lo-Mendell-Rubin Likelihood Ratio Test (LMRT) and Bootstrap Likelihood Ratio Test (BLRT) were less than 0.05, and classes containing at least 5% of the sample were considered valid. Model selection also considered substantive interpretability.

Differences among latent profiles were examined using Chi-squared (χ^2^) tests and one-way ANOVA (*p* < 0.05). Significant factors were then entered into multivariate logistic regression (α = 0.05). Finally, the mediating role of PCS between PsyCap and bullying experiences was tested using the PROCESS macro (Model 4) with 5,000 bootstrap samples. All variables were standardized in advance. A 95% bias-corrected confidence interval that did not include zero suggested significant mediation. All two-tailed tests were set at a significance threshold of *p* < 0.05.

## 3 Results

### 3.1 Participant characteristics

Of the 578 participants in this study, 433 were female, accounting for 74.9%. The mean age was 22.03 ± 2.23 years, and most held a bachelor's degree or higher. In terms of internship duration, approximately half of the interns had completed 6–10 months of clinical practice. Regarding character features, approximately half of the students self-identified as introverted (51.9%). Based on self-assessment, most students perceived their relationship with their instructors as “neutral”. Detailed participant characteristics can be found in Table 1.

**Table 1:** Differences in demographic and study variables across latent profiles (*N* = 578).

Characteristic	*N* (%)/mean ±SD	Class 1	Class 2	Class 3	χ^2^/*F*	*p*
		***N*** **(%)/mean** ±**SD**	***N*** **(%)/mean** ±**SD**	***N*** **(%)/mean** ±**SD**		
Age (year, mean ± SD)	22.03 ± 2.23	22.47 ± 2.14	21.84 ± 2.43	21.86 ± 2.13	4.617^b^	0.017
Gender					27.000^a^	< 0.001
Male	145 (25.1)	25 (14.8)	63 (39.1)	57 (23.0)		
Female	433 (74.9)	144 (85.2)	98 (60.9)	191 (77.0)		
Education background					43.690^a^	< 0.001
Junior college	147 (25.4)	54 (32.0)	16 (9.9)	77 (31.0)		
Undergraduates	311 (53.8)	62 (39.6)	115 (71.4)	129 (52.0)		
Postgraduates	120 (20.8)	48 (28.4)	30 (18.6)	42 (16.9)		
Internship duration (months)					35.700^a^	< 0.001
3 to 6	139 (24.0)	24 (14.2)	57 (35.4)	58(23.4)		
6 to 10	328 (56.7)	112 (66.3)	62 (38.5)	154(62.1)		
More than 10	111 (19.2)	33 (19.5)	42 (26.1)	36(14.5)		
Character features					31.312^a^	< 0.001
Introvert	295 (51.0)	56 (33.1)	90 (55.9)	149(60.1)		
Extrovert	283 (49.0)	113 (66.9)	71 (44.1)	99(39.9)		
Relationship with instructors					25.350^a^	< 0.001
Most were harmonious	184 (31.8)	62 (36.7)	46 (28.6)	76 (30.6)		
Most were neutral	348 (60.2)	100(28.6)	88 (54.7)	160 (64.5)		
Most were discordant	46 (8.0)	7 (30.6)	27 (16.8)	12 (4.8)		
Psychological capital	78.44 ± 17.22	91.19 ± 12.08	63.99 ± 15.11	79.13 ± 14.06	160.190^b^	< 0.001
Self-efficacy	23.49 ± 5.89	27.46 ± 4.11	18.63 ± 5.15	23.93 ± 5.09	138.878^b^	< 0.001
Hope	23.43 ± 5.59	27.36 ± 4.06	19.09 ± 5.18	23.58 ± 4.64	130.823^b^	< 0.001
Resilience	19.63 ± 4.74	22.76 ± 3.73	16.17 ± 4.36	19.73 ± 4.03	109.726^b^	< 0.001
Optimism	11.89 ± 2.92	13.61 ± 2.43	10.09 ± 2.74	11.89 ± 2.62	75.732^b^	< 0.001
Perceptions of clinical stressors	81.59 ± 20.54	63.07 ± 13.20	98.58 ± 15.52	80.86 ± 16.65	218.544^b^	< 0.001
Instructor's limited clinical competence	16.64 ± 4.54	13.05 ± 3.84	19.86 ± 3.56	17.09 ± 3.73	139.265^b^	< 0.001
Inadequate clinical environment	21.21 ± 6.29	16.05 ± 4.68	26.19 ± 4.67	21.58 ± 5.40	171.296^b^	< 0.001
Interns' inadequate knowledge and skills	8.77 ± 2.56	7.06 ± 2.32	10.40 ± 1.99	8.93 ± 2.33	93.201^b^	< 0.001
Inefficient clinical teaching planning	11.07 ± 3.28	8.51 ± 2.64	13.29 ± 2.74	8.51 ± 2.20	128.495^b^	< 0.001
Instructors' inappropriate teaching behavior	13.09 ± 3.83	9.98 ± 2.51	15.88 ± 3.38	13.44 ± 3.31	150.168^b^	< 0.001
Concerns about nursing career characteristic	10.81 ± 3.34	8.40 ± 2.54	12.96 ± 3.04	11.14 ± 2.98	106.686^b^	< 0.001

Class 1, low bullying group; Class 2, high bullying group; Class 3, moderate bullying group. ^a^Chi-square test.^b^ One-way analysis of variance (ANOVA).

### 3.2 Common method bias test and correlation analysis

Harman's single-factor test was conducted to examine common method bias. The results indicated that 8 factors had eigenvalues greater than 1. The first factor accounted for 37.86% of the total variance, which was below the recommended threshold of 50%. Therefore, common method bias was not a serious concern in this study. Correlations for the main variables are presented in Table 2. Means and standard deviations were as follows: PsyCap (M = 78.44, SD = 17.22), PCS (M = 81.59, SD = 20.54), and bullying experiences (M = 21.22, SD = 15.59). Pearson correlations analysis revealed a significant correlation between PsyCap and bullying experiences (*r* = −0.635, *p* < 0.001). PsyCap was negatively correlated with PCS (*r* = −0.558, *p* < 0.001), whereas PCS positively correlated with bullying experiences (*r* = 0.692, *p* < 0.001).

**Table 2:** Correlations among study variables.

Variables	1	2	3
1. PsyCap	1		
2. PCS	−0.564^***^	1	
3. BE	−0.651^***^	0.655^***^	1

^*^p < 0.05; ^**^p < 0.01; ^***^p < 0.001. SD, standard deviation. PsyCap, psychological capital; PCS, perceptions of clinical stressors; BE, bullying experiences.

### 3.3 Latent profiles model selection and naming of bullying experiences among nursing students

As shown in Table 3, AIC, BIC, and aBIC decreased with the increase in the number of categories. The rate of decrease slowed from 2 to 4 categories and became more gradual, with all Entropy values remaining above 0.9. Additionally, the LMR value for the four-category model was not significant, indicating that it did not outperform the three-category model. Furthermore, considering model accuracy and to avoid misclassification, each subgroup should contain at least 10% of the sample size (34). Therefore, we selected the three-category model as optimal. The average posterior probabilities for the 3 classes were 0.958, 0.986, and 0.957, respectively.

**Table 3:** Latent profile model fit information for bullying experiences.

Model	Loglikelihood	AIC	BIC	aBIC	Entropy	LMR(*p*)	BLRT(*p*)	Class Probabilities (%)
1	−6,555.948	13,127.896	13,162.773	13,137.376	-	-	-	100
2	−5,796.554	11,619.108	11,675.783	11,634.513	0.949	< 0.001	< 0.001	68.3/31.7
**3**	–**5,526.877**	**11,089.755**	**11,168.227**	**11,111.085**	**0.922**	**0.0005**	<**0.001**	**29.2/42.9/27.9**
4	−5,353.477	10,752.953	10,853.224	10,780.208	0.937	0.0546	< 0.001	29.1/25.1/41.7/4.2

AIC, akaike information criterion; BIC, Bayesian information criterion; aBIC, sample-size adjusted Bayesian information criterion; LMR, Lo-Mendell-Rubin likelihood ratio test; BLRT, bootstrap likelihood ratio test. Bold values indicate the optimal model selected based on the best fit indices (i.e., the 3-class model), which was determined by the lower AIC, BIC, and aBIC values, the higher Entropy (>0.90), and significant LMR and BLRT tests (*p* < 0.05).

**Figure 1:**
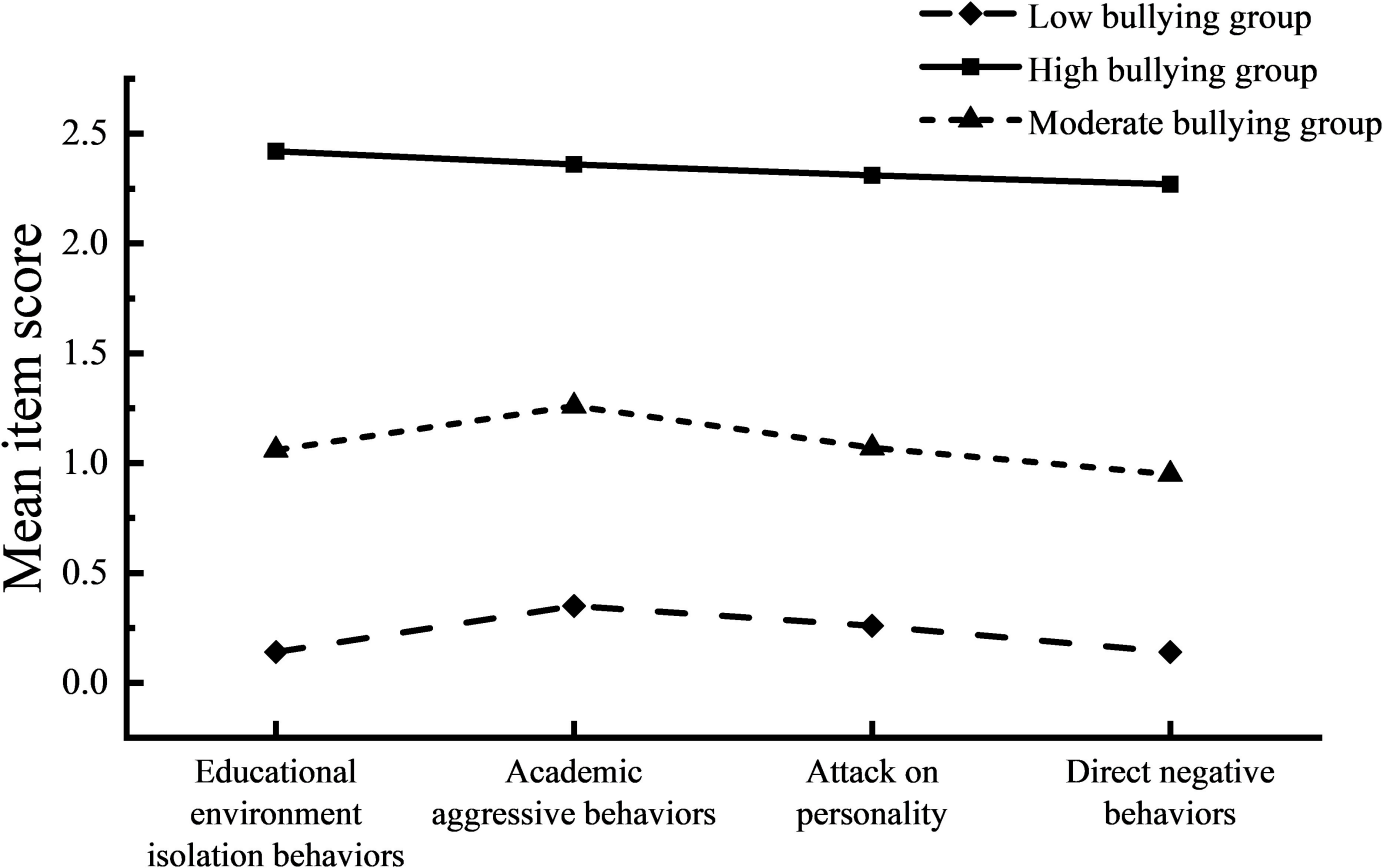
Mean item scores of the three latent profiles across the four dimensions of bullying experiences. Class 1, low bullying group (*n* = 169); Class 2, High bullying group (*n* = 161); Class 3, Moderate bullying group (*n* = 248). The four dimensions are: educational environment isolation behaviors, academic aggressive behaviors, attack on personality, and direct negative behaviors.

Figure 1 illustrates the mean item scores of the three latent classes across the four dimensions of bullying experiences. Class 1 comprised 169 nursing students (29.2%), whose average scores on the four dimensions—educational environment isolation behaviors, academic aggressive behaviors, attack on personality, and direct negative behaviors—were 0.56, 1.41, 1.53, and 0.56, respectively, suggesting minimal bullying experiences. Accordingly, this class was named the “Low Bullying Group”. Class 2 consisted of 161 students (27.9%) and their average scores on the four dimensions were 9.66, 9.45, 13.86, and 9.07, respectively, indicating frequent bullying experiences across all dimensions. Thus, this class was named the “High Bullying Group”. Class 3 comprised 248 students (42.9%), with average scores of 4.25, 5.03, 6.41, and 3.80, respectively, all at moderate levels between the other two groups. This class was therefore named the “Moderate Bullying Group.”

### 3.4 Distributional characteristics of latent profiles of bullying experiences among nursing students

Analysis of variance and chi-square tests revealed significant differences across all variables among the three groups (all *p* < 0.05). Specifically, the low bullying group had the highest PsyCap scores and the lowest PCS scores, whereas the high bullying group showed the opposite pattern, with the moderate bullying group falling in between. In terms of demographic characteristics, the high bullying group had a higher proportion of male (39.6%) and introverted students (55.0%), as well as more students in the early internship stage (35.5%), compared to the other two groups. Detailed results are presented in Table 1.

### 3.5 Multivariate logistic regression analysis of latent profiles of bullying experiences among nursing students

Multivariate logistic regression analysis was conducted to identify predictors associated with the likelihood of classification into each latent class, with the low bullying group as the reference (Table 4). Age, education background, and relationship with instructors were not significant predictors. Male participants were more likely than females to belong to the high bullying group (OR = 4.428, *p* = 0.001) or moderate bullying group (OR = 2.293, *p* = 0.021). Introverted students were more likely than extroverted students to be classified into the moderate bullying group (OR = 2.577, *p* < 0.001). Students in the early internship stage (3–6 months) were more likely to be classified into the moderate bullying group (OR = 3.232, *p* = 0.023) than those with longer internships. In addition, PsyCap and PCS were significant for the high bullying group (OR = 0.894, *p* < 0.001; OR = 1.157, *p* < 0.001) and moderate bullying group (OR = 0.950, *p* < 0.001; OR = 1.095, *p* < 0.001).

**Table 4:** Results of multivariate logistic regression analysis (*N* = 578).

**Variable**	Class 2 vs. Class 1							Class 3 vs. Class 1				
	***B***	***SE***	***Wald***	***OR***	***95%CI***	***p***	***B***	***SE***	***Wald***	***OR***	***95%CI***	***p***
Age	−0.196	0.104	3.586	0.822	[0.671, 1.007]	0.058	−0.113	0.085	1.744	0.893	[0.756, 1.056]	0.187
Gender												
Male	1.488	0.437	11.578	4.428	[1.879, 10.432]	**0.001**	0.830	0.360	5.323	2.293	[1.133, 4.642]	**0.021**
**Education background (Postgraduates as ref)**												
Junior college	−1.055	0.707	2.225	0.348	[0.087, 1.393]	0.136	0.429	0.534	0.644	1.535	[0.539, 4.376]	0.422
Undergraduates	0.181	0.538	0.113	1.199	[0.417, 3.444]	0.736	0.222	0.432	0.264	1.249	[0.535, 2.912]	0.607
Internship duration (more than 10 months as ref)												
3–6 months	1.166	0.600	3.777	3.209	[0.990, 0.400]	0.052	1.173	0.516	5.171	3.232	[1.176, 8.884]	**0.023**
6–10 months	−0.324	0.502	0.416	0.723	[0.271, 1.934]	0.519	0.386	0.416	0.865	1.472	[0.652, 3.324]	0.352
**Character features (Extrovert as ref)**												
Introvert	0.550	0.358	2.355	1.732	[0.859, 3.495]	0.125	0.947	0.272	12.143	2.577	[1.513, 4.390]	**< 0.001**
**Relationship with instructors (Most were discordant as ref)**												
Most were harmonious	−0.557	0.761	0.535	0.573	[0.129, 2.547]	0.465	0.721	0.675	1.140	2.056	[0.548, 7.721]	0.286
Most were neutral	−1.003	0.717	1.955	0.367	[0.090, 1.496]	0.162	0.278	0.649	0.183	1.320	[0.370, 4.712]	0.669
**PsyCap**	−0.112	0.015	59.493	0.894	[0.869, 0.920]	**< 0.001**	−0.051	0.011	20.588	0.950	[0.930, 0.972]	**< 0.001**
**PCS**	0.146	0.014	102.854	1.157	[1.125, 1.191]	**< 0.001**	0.090	0.011	69.636	1.095	[1.072, 1.118]	**< 0.001**

PsyCap, psychological capital; PCS, perceptions of clinical stressors; Class 1, low bullying group; Class 2, high bullying group; Class 3, Moderate bullying group. Bold values indicate statistically significant results in the multivariate logistic regression analysis (*p* < 0.05).

### 3.6 Mediation analysis of PCS

**Figure 2:**
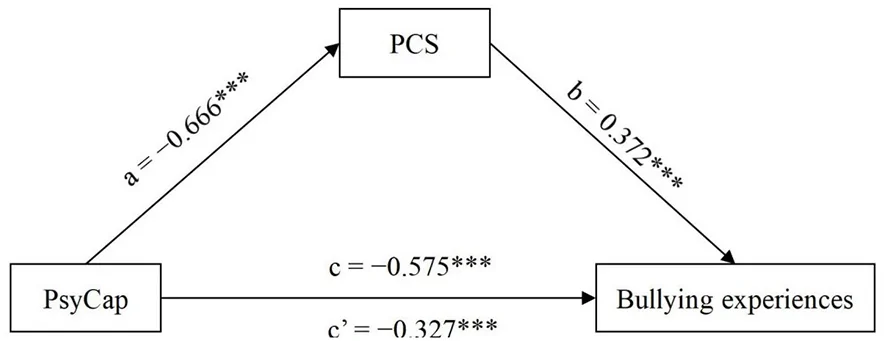
Mediation model of perceptions of clinical stressors. ***p < 0.001. PsyCap, psychological capital; PCS, perceptions of clinical stressors.

After adjusting for age, gender, education background, internship duration, character features, PsyCap significantly predicted PCS in a negative direction (*a* = −0.666, SE = 0.041, *p* < 0.001). When both PsyCap and PCS were included in the model, both significantly predicted bullying experiences (*c'* = −0.327, SE = 0.030, *p* < 0.001; *b* = 0.372, SE = 0.025, *p* < 0.001). The total effect of PsyCap on bullying experiences was *c* = −0.575 (SE = 0.029, *p* < 0.001), with detailed results presented in Table 5. The bootstrap analysis confirmed that PCS significantly mediated the relationship between PsyCap and bullying experiences (*ab* = −0.248, BootSE = 0.025, 95% CI [−0.300, −0.201]), with a proportion mediated of 43.12% (*ab*/*c* = 43.12%). These findings suggest that PCS partially mediated the relationship between PsyCap and bullying experiences, as illustrated in Figure 2.

**Table 5:** Analysis of the effects of PsyCap on bullying experiences.

**Effect**	Path	B	SE	*t*	*P*	95%CI
Path *a*	PsyCap → PCS	−0.666	0.041	−16.143	< 0.001	
Path *b*	PCS → BE	0.372	0.025	14.844	< 0.001	
Direct effect (*c'*)	PsyCap → BE	−0.327	0.030	−10.925	< 0.001	[−0.385, −0.268]
Indirect effect (*ab*)	PsyCap → PCS → BE	−0.248	0.025	—	—	[−0.300, −0.201]
Total effect (*c*)	PsyCap → BE	−0.575	0.029	−19.702	< 0.001	[−0.632, −0.517]

PsyCap, psychological capital; PCS, perceptions of clinical stressors; BE, bullying experiences. Bootstrap samples, 5000. 95% CI = 95% confidence interval.

## 4 Discussion

Prior studies on nursing student clinical bullying have predominantly adopted variable-oriented approaches, often overlooking population heterogeneity and complex variable relationships. Drawing on the conservation of resources (COR) theory, this study integrated person-centered latent profile analysis and variable-based mediation modeling. We identified three distinct profiles of bullying experiences with different severity levels, characterized subgroup attributes, and found that PCS mediated the relationship between PsyCap and bullying experiences. Collectively, these results provide actionable implications for nursing clinical educators and for maintaining stability within the nursing workforce. All research hypotheses were supported by the current data.

Three distinct profiles of bullying experiences were identified: the “Low bullying group” (29.2%), the “High bullying group” (27.9%), and the “Moderate bullying group” (42.9%). These findings supported Hypothesis 1, confirming significant heterogeneity in how nursing students experience bullying. The moderate bullying group, which constituted the largest proportion of participants, implies that most nursing students are situated in a vulnerable intermediate state. These students face diffuse moderate-level bullying across multiple dimensions but have not developed effective long-term coping strategies. Under the cumulative impact of adverse clinical stimuli, this group is at high risk of deteriorating into severe victimization, revealing the latent risk prevailing in clinical education (35). Notably, students in the high bullying group experienced high levels across all four dimensions of bullying. This pattern suggests that these students are exposed to pervasive, multidimensional victimization rather than being targeted through a single type of behavior. The high bullying profile likely reflects entrenched negative dynamics in the clinical environment, such as hierarchical marginalization by staff and preceptors who view students as temporary auxiliary labor. According to COR theory, interpersonal connection and supportive learning atmosphere constitute important social resources for individuals (36). Persistent, multi-dimensional bullying depletes such social resources continuously, cuts off students' access to guidance and emotional support, and eventually forms a vicious cycle of “resource loss → increased vulnerability to bullying”.

Multinomial logistic regression further revealed that male students, introverted individuals, and those at the early stage of internship (3–6 months) were more likely to belong to the moderate or high bullying group. In predominantly female nursing environments, male students may experience heightened visibility and role incongruence (37), which may increase their susceptibility to social exclusion and marginalization (38). Introversion probably restricts active social engagement and help-seeking behaviors; combined with the hierarchical culture in clinical departments, introverted students were 2.577 times more likely to be classified into the high bullying group than extroverted peers. The higher risk observed in introverted individuals should be understood as an interaction between personality traits and unsupportive educational environments, rather than as a personality deficit per se (39). Students in the early internship stage (3–6 months) were 3.232 times more likely to be classified into the moderate bullying group, suggesting that the initial adaptation period of clinical practice is a critical window for stress accumulation and bullying experiences. The high bullying subgroup represents the high-risk population that requires priority intervention in clinical management. Comparatively, the low bullying group maintains relatively complete psychological and social resources and forms adaptive interaction patterns with clinical teams, which can be regarded as a positive example of clinical adaptation.

The findings verified a significant negative correlation between PsyCap and bullying experiences (*r* = −0.635, *p* < 0.001), which is consistent with Hypothesis 2. This result is generally in line with the study by Hwang (40), which suggested that PsyCap serves as a crucial protective factor against psychological distress, promotes personal growth and buffers the negative impacts of high clinical demands. From the perspective of COR theory, PsyCap functions as an individual's internal psychological resource reservoir, which compensates for resource loss caused by negative events, alleviates emotional exhaustion, and strengthens psychological defense against adverse behaviors. Therefore, these results not only validate the strong explanatory power of COR theory but also expand the practical application scope of this theoretical framework. Although this cross–sectional study cannot verify causal pathways, cultivating nursing students' PsyCap may serve as a protective factor against bullying experiences. Evidence suggests that targeted micro-intervention training derived from the Psychological Capital Intervention Model, which focuses on fostering hope, self-efficacy, resilience and optimism, can effectively improve individual PsyCap (41). Moreover, positive psychology interventions such as mindfulness-based stress reduction training, constructive competitive activities, positive guidance from clinical instructors, and job crafting are potentially conducive to creating safer clinical learning environments (23, 42).

Hypothesis 3 was also supported with a significant indirect effect, confirming that PCS partially mediates the relationship between PsyCap and bullying experiences (*ab* = −0.248, BootSE = 0.025, 95% CI [−0.300, −0.201]), with a proportion mediated of 43.12%. In the mediation model, the standardized direct effect of PsyCap on bullying experiences after controlling for PCS was −0.327 (*p* < 0.001), and the standardized total effect was −0.575 (*p* < 0.001). These results suggest that PCS may attenuate the protective effect of PsyCap, a finding consistent with the Stress and Coping Model, which recommends multiple coping strategies focusing on improvement of stress appraisal (43). High PsyCap may enhance nursing students' abilities to manage stress and maintain mental health, which aligns with prior research (44) and further supports the role of PsyCap in stress perception. According to COR theory, clinical stressors represent chronic stress stimuli, sustained high stress may gradually erode nursing students' resource reserves, weakening their capacity to resist inappropriate words and behaviors from others. In addition, inadequate clinical teaching environments, marked by limited guidance and ambiguous role expectations (45, 46), may further exacerbate feelings of isolation by restricting meaningful integration into clinical teams. Furthermore, excessive concerns about nursing career characteristics may signal uncertainty regarding professional identity and long–term career matching (47), heightening sensitivity to interpersonal stressors. This pattern appears more prominent among students in the high bullying group, which could be attributable to low psychological resilience and insufficient positive coping styles. Therefore, integrating psychological resource cultivation (i.e., PsyCap) and cognitive appraisal adjustment (i.e., PCS) into clinical teaching and management is a plausible strategy to strengthen nursing students' capacity to resist bullying and alleviate its adverse effects.

In summary, LPA analysis revealed significant heterogeneity in bullying experiences among nursing students. Furthermore, a negative association was observed between PsyCap and bullying experiences, with PCS serving as a partial mediator in this association. Drawing on these findings, we propose targeted tiered interventions based on subgroup classification. For the low bullying group, educators should consolidate their adaptive coping modes and maintain their positive resource states. Meanwhile, standardized pre–internship training on bullying recognition and coping skills should be incorporated into the curriculum to build an early risk prevention barrier before nursing students participate in clinical rotations. For the dominant moderate bullying group, the core task is to implement universal stress reduction. Structured mentorship programs, explicit role clarification for students, and accountability mechanisms for clinical instructors, coupled with replacing incompetent instructors and optimizing feedback channels, may jointly reduce students' perceptions of bullying experiences. For the high bullying group, interventions must combine environmental governance with individual resource cultivation. On the one hand, establishing transparent bullying reporting channels and intervention mechanisms can help break the isolated learning atmosphere. On the other hand, targeted PsyCap training and interpersonal competency development, especially for introverted and male students, can improve their initiative of communication and help-seeking. To translate these findings into practice, clinical educators can use the 18-item BBNE scale as a screening tool at the midpoint of clinical rotations (approximately 3–4 months into the internship). Students whose score patterns match the low bullying profile can be assigned to routine monitoring; those matching the moderate profile can be offered structured mentorship and monthly debriefing sessions; and those matching the high profile can be referred for individualized support, including one-on-one counseling and facilitated communication with clinical instructors. This screening-based approach does not require complex statistical modeling and can be feasibly integrated into routine clinical supervision. In summary, through the identification of distinct characteristics of bullying experiences in nursing students, educators can proactively implement timely interventions to support vulnerable students and address bullying experiences in clinical learning environments.

### 4.1 Limitation

Several limitations of this study should be acknowledged. First, the cross-sectional design precludes causal inference. The resource depletion process reflected in the mediation model is theoretically deduced rather than empirically verified by longitudinal data. Future longitudinal panel studies could track the dynamic changes of PsyCap, PCS and bullying experiences across different internship stages to reveal causal sequences and resource-loss trajectories. Second, participants were recruited only from tertiary general hospitals in Gansu Province. Differences in hospital hierarchy, regional cultural atmosphere and educational background may affect students' stress perception and bullying experiences, limiting the generalizability of the conclusions. Subsequent research could expand sampling scope and conduct regional comparative analyses. Third, this study focused primarily on individual psychological factors. Organizational factors such as ward atmosphere, team culture and management regulations are also important antecedents of clinical bullying. Follow-up studies could integrate multi-level variables to construct more comprehensive predictive models. Fourth, although Harman's single-factor test suggested that common method bias was not a serious concern, future research should consider more rigorous methods [e.g., CFA marker techniques (48)] to address common method variance. Fifth, the classification of students' personality as introverted or extroverted relied on a single self-report item; this approach, while pragmatic, does not capture the full nuance of personality structure and may be subject to reporting biases. Moreover, measurement invariance across gender was not tested, so differences between male and female students should be interpreted with caution until it is established that the BBNE functions equivalently in both groups. Finally, introverted students may perceive and report interpersonal stressors more sensitively, which could inflate bullying experience scores independent of objective exposure.

## 5 Conclusion

This study employed LPA to reveal the heterogeneous characteristics of bullying experiences among nursing students, identifying three latent categories: “Low bullying group” (29.2%), “High bullying group” (27.9%), and the “Moderate bullying group” (42.9%). Findings indicate a significant negative association between PsyCap and bullying experiences, with PCS partially mediating this relationship. This study expands the application of COR theory in the field of nursing education bullying research, and provides person-centered empirical evidence for developing precise intervention strategies. To safeguard nursing students' physical and mental health and stabilize the nursing talent reserve, clinical educators and managers should fully recognize group heterogeneity, combine organizational environment optimization with individual psychological capital intervention, and build a secure and supportive clinical learning environment.

## Data Availability

The raw data supporting the conclusions of this study will be made available by the authors upon reasonable request, without undue reservation.
